# Distribution of Recessive Genetic Defect Carriers in Holstein Friesian Cattle: A Polish Perspective

**DOI:** 10.3390/ani14223170

**Published:** 2024-11-06

**Authors:** Marta Gozdek, Sebastian Mucha, Adam Prostek, Dariusz Kamola, Tomasz Sadkowski

**Affiliations:** 1 Department of Physiological Sciences, Institute of Veterinary Medicine, Warsaw University of Life Sciences, 02-776 Warsaw, Poland; marta_gozdek@sggw.edu.pl (M.G.); adam_prostek@sggw.edu.pl (A.P.); 2 Polish Federation of Cattle Breeders and Dairy Farmers, 00-515 Warsaw, Poland; s.mucha@cgen.pl; 3 The Kielanowski Institute of Animal Physiology and Nutrition, Polish Academy of Sciences, 05-110 Jabłonna, Poland; dariuszkamola@gmail.com

**Keywords:** Holstein Friesian, genetic disease, recessive mutation, genotyping

## Abstract

Genetic disorders are caused by a hereditary change in the structure of DNA that may hurt the health and life of animals. Several recessive haplotypes and a few causative mutations are known in Holstein Friesian cattle: CDH (Holstein cholesterol deficiency), haplotypes with a homozygous deficiency in Holstein (HH1, HH3, HH4, HH5, HH6, HH7), BLAD (bovine leukocyte adhesion deficiency), DUMPS (deficiency of uridine monophosphate synthase), FXI (factor XI deficiency), HHM (mule foot, syndactyly), and BC (citrullinaemia). From a breeding point of view, these genetic diseases have highly negative effects and are a significant problem for breeders, exposing them to economic losses and hurting animal welfare. This study aimed to characterize the Polish population of Holstein Friesian dairy cattle, considering the carrier status of twelve selected genetic defects. This study was based on genotype data collected from 78,884 cows and 691 bulls of the Holstein Friesian variety. The studies were performed using Illumina Infinium microarrays. Among both bulls and cows, the highest numbers of carriers were detected for HH5 (appropriately 6.7% and 5.4%). The lowest numbers of carriers were detected for DUMPS, factor XI, and HHM. The study revealed one calf suffering from cholesterol deficiency.

## Simple Summary

There are several known genetic diseases affecting Holstein Friesian cattle. The vast majority of them are lethal; they cause the death of the sick animal in the fetal period or shortly after birth. These genetic disorders cause multiple problems for breeders, exposing them to economic losses, e.g., treatment costs, and repeated inseminations. The aim of the present study was to determine the frequency of the occurrence of twelve selected genetic diseases in the Polish population of Holstein Friesian dairy cattle. A total of 78,884 cows and 691 bulls were genotyped by Illumina microarrays. Our results showed a relatively high frequency of some genetic diseases in the Polish population of Holstein Friesian cattle. The results obtained clearly highlight the need to take action to eliminate these genetic diseases from the Holstein Friesian cattle population, thus reducing losses in the fertility, productivity, and profitability of dairy cattle enterprises in the future.

## 1. Introduction

Genetic mutations are changes in the sequence of the coding or the regulatory regions of genes. Gene mutations can be beneficial, such as *DGAT1* polymorphisms, which have significant effects on milk yield, fat yield, protein yield, and fat and protein contents. On the other hand, mutations can also have an adverse effect by causing genetic diseases. It has been proven that even a single change in the DNA sequence can contribute to disturbances in the production of functional protein or in the gene expression level, changing the amount of protein produced [1]. Usually, genetic disorders are typically infrequent and impact one animal in every several thousand or million individuals. The majority of these disorders manifest sporadically and are of limited significance, but some increase in frequency to the point that they become a significant economic concern and need to be monitored and selected against [2,3]. In cattle breeding, genetic disorders are an important issue, gaining increasing interest from breeders.

The appearance of genetic defects in farm animals, manifested by morphological disorders or the impairment of various body functions (e.g., immune, metabolic, etc.), is a common cause of animal diseases and even causes the death of sick animals. Some genetic diseases affect fertility due to increased embryonic mortality. Calf morbidity and mortality are associated with high costs for the farmer, such as the costs of medical treatment, economic losses arising from extended service periods and calving intervals, and premature culling due to infertility [4]. A loss of fertility due to Holstein haplotypes (HHs) has direct consequences on the management costs of the herd due to the increase in calving interval and the decrease in the conception rate.

The most common inheritance pattern of a genetic disease is a simple recessive trait. If both parents are heterozygous for a defective recessive allele, 25% of their progenies are expected to be homozygous recessive and thus express the disease phenotype [2,3]. The phenotype of a heterozygous carrier of the disease does not differ from the phenotype of healthy animals. This makes it much more difficult to recognize the disease early and take preventive measures that could prevent the economic losses incurred by breeders, resulting from the deaths of sick animals or their poorer production results. One of the factors increasing the frequency of genetic diseases is the “founder effect”—the emergence of genetic diseases in new populations derived from small numbers of animals of noble breeds (“founder” animals) through mating them with each other. The Holstein Friesian cattle breed is a genetically small population formed a few centuries ago from a limited number of founders [5,6]. A factor that contributes to the “spread” of genetic diseases is low individual variability within a given population or breed. Over the last 60 years, the gene pool of the Holstein Friesian cattle population was reduced by the widespread use of a limited number of elite sires via artificial insemination, the international trading of semen and embryos, and strong selection based on a limited number of traits [5]. Such intensive selection led to an increase in inbreeding, which translated into an increase in homozygosity and created favorable conditions for the expression of recessive defects [7]. An additional factor that makes it difficult to eliminate genetic defects from a population is the frequent combination of a high breeding value of an animal with a hidden genetic defect [8]. An example would be Pennstate Ivanhoe Star, who was the common ancestor of bulls carrying CVM (complex vertebral malformation) and BLAD (bovine leukocyte adhesion deficiency). These 2 defects were mainly spread throughout the world by his son, Carlin-M Ivanhoe Bell [9]. Above all, however, the widespread use of the artificial insemination method has been a significant factor influencing already consolidated breeds, especially among Holstein Friesian cattle. It has made it possible to obtain thousands of offspring from a single bull in the entire world population. As a result of the globalization of animal breeding, the spread of mutations is occurring in many countries at a very rapid pace.

Genetic diseases occur in all breeds of cattle; however, some defects are strongly associated with certain breeds. The increasing prevalence of hereditary anomalies in the Holstein Friesian cattle population presents a pressing issue—caused by embryonic mortality and the birth of non-viable offspring. In recent decades, the genetic basis of several lethal defects in Holstein Friesian cattle has been identified, including BLAD [10]; Holstein haplotypes such as HH1 [11], HH3 [12], HH4 [5], HH5 [13], HH6 [14], and HH7 [15]; DUMPS [16]; Holstein cholesterol deficiency (CDH) [17]; factor XI deficiency (FXI) [18]; HHM (mule foot, syndactyly) [19]; and citrullinaemia (BC) [20] (Table 1).

HH1, HH3–HH7, and DUMPS are caused by recessive mutations that cause embryo death at any stage of pregnancy. Miscarriages occur at less than 35 days of gestation for HH6 and HH7, at around 40 days of gestation for DUMPS, between 60 and 100 days of gestation for HH3, HH4, and HH5, and at all stages of gestation for HH1. BLAD causes a decrease in the immunity of calves, leading to recurrent respiratory and digestive system infections and, consequently, to poorer growth and development, usually leading to the death of the animals. Calves affected by CDH are underdeveloped in terms of weight and show progressive and severe emaciation, despite normal feed intake. These calves show secondary diseases and symptoms, such as pneumonia. Calves affected by BLAD and CDH are born but die within a few days due to severe diarrhea and infections [21]. Calves affected by citrullinaemia are unable to excrete ammonia and display neurological symptoms that become progressively worse, leading to death within one week of birth [22]. Mule foot is a genetic disorder that constitutes the non-division or fusion of digits, and it mostly appears as the synostosis of phalanges. This disease might affect only one foot or all four feet. However, two gradients are observed: front–rear and right–left [23]. FXI deficiency is a rare genetic coagulopathy of cattle that occurs worldwide due to a deficiency in factor XI, an important protein in the coagulation cascade. Calves with factor XI deficiency can have varying lifespans. This depends on many factors, such as the quality of veterinary care, husbandry conditions, and the individual health of the calf. Calves can live normal lives with proper management and treatment [24]. The genetic defects analyzed in this article have been described by Gozdek et al. (2024) [21].

The first defect identified in Poland, based on DNA, was DUMPS. Research on this has been conducted since 1995 at the University of Warmia and Mazury (UWM) research center in Olsztyn. As part of this research, 2209 bulls approved for breeding were examined over a period of 9 years. No DUMPS carrier was found [25]. In 1995, the Olsztyn Center began identifying carriers of BLAD, and since 1999, black-and-white and red-and-white bulls intended for breeding have been subjected to mandatory BLAD tests [26].

All the genetic defects examined in our study are found on the Illumina microarray, which is used for the genomic evaluation of cattle worldwide, including in Poland. Since 2021, the Polish Federation of Cattle Breeders and Dairy Farmers has identified all the genetic defects described in our study. The problem of genetic defects cannot be underestimated because it is unknown how many carriers of genetic defects occur in the Polish population of Holstein Friesian cattle. By monitoring carriers of these defects in both females and males and by appropriately selecting animals for mating, it is possible to gradually eliminate the occurrence of genetic defects in the population while simultaneously achieving genetic progress.

This study aimed to characterize the Polish population of Holstein Friesian dairy cattle, considering the carrier status of twelve selected genetic defects.

## 2. Materials and Methods

Following Resolution No. 13/2016 of the National Ethics Committee for Animal Experiments (Poland) of 17 June 2016, the Ethics Committee’s consent is not required to collect animal material for genotyping. This study was carried out on 691 bulls and 78,884 Polish black-and-white Holstein Friesian dairy cows born between 2020 and 2024. The genetic material for the study was collected from the ear punch during the routine estimation of breeding value (EBV). Genotyping was performed through DNA microarrays by the Cattle Genetics Laboratory of the Polish Federation of Cattle Breeders and Dairy Farmers (PFCB&DF, Warsaw, Poland). As a part of Euro Genomics, cooperative PFCB&DF uses customized EuroGenomics arrays by utilizing the version called EuroGenomics MD_POL. The following arrays were used in the present study: EuroGenomics_MD_v2_POL, EuroGenomics_MD_v3_POL, EuroGenomics_MD_v4_POL, and EuroGenomics_MD_v4-1_POL. The number of cows and bulls genotyped on individual microarrays is shown in Table 2.

### 2.1. DNA Isolation

Biopsy samples (ear punch) were collected using the Allflex Tissue Sampling Unit (Merck & Co., Dallas, TX, USA) during the period from October 2021 to June 2024. Samples were kept at room temperature and transferred to the laboratory by post after a few days. Samples were coded and transferred to 96-well plates, after which the lysis buffer and proteinase K were added. This mix was then incubated at 56 °C and mixed (600 RPM) in a thermomixer overnight, followed by centrifugation at 10,000 RPM for 2 min to remove undigested parts. Next, the lysate was transferred to deep-well plates, and DNA extraction was performed using the following kits: we used the (a) Clean Blood & Tissue DNA Kit (CleanNA, Waddinxveen, Nederlands) according to the manufacturer’s protocol in the KingFishre Duo DNA system (Thermo Fisher, Waltham, MA, USA) and the (b) MagnifiQ™ 1 Genomic DNA instant kit (A&A Biotechnology, Gdańsk, Poland) according to the producer manual in the Nucleic Acid Extractor BNP48 (BIOBASE, Jinan, China).

### 2.2. Microarrays

DNA, normalized to 50 ng/µL, was processed according to the Illumina Infinium XT protocol (manual protocol). Beadchips were immediately scanned on the Illumina iScan system and analyzed using GenomeStudio Software V2011.1 version 2.1 (Illumina, San Diego, CA, USA). All samples from one type of array were again clustered together before export using a cluster file. Only samples with call rates over 0.95 were chosen for subsequent statistical analysis. The EuroGenomics arrays’ content (probe list and sequences) is confidential and has underlying restrictions in terms of publication. Only the SNPs present in all array versions were selected for further analysis. Farmers authorized the authors to access a portion of the SNP data. The SNP data for the genes tested in the present study are presented in Table 3.

### 2.3. Statistical Analysis

The percentage of carriers was determined based on the formula:

a/b ∗ 100

where “a” is the number of carriers of the genetic defect and “b” is the total number of animals tested.

## 3. Results

### 3.1. Cows

A total of 78,884 Polish Holstein Friesian cows were assessed in this study. The frequencies of carriers for each locus are given in Table 4. Overall, the highest carrier frequency was observed for HH5 (5.40%), while the lowest carrier was found for FXI (<0.01%).

During the study period, only one animal was found to have cholesterol deficiency. The affected calf, born without any apparent abnormalities, was underdeveloped, especially in terms of weight. The affected calf did not respond to any medical treatment and showed progressive emaciation despite normal feed intake. After 6 weeks, the animal died.

Overall, 17.48% of cows (13,793) were carriers of one genetic defect. Double and triple genetic defects were carried by 854 and 11 cows, respectively. One animal carried four genetic defects. The remaining 12,927 cows carried a single genetic defect (Table 5). In the case of animals with a double genetic defect, the research conducted showed that the most common occurrences were as follows: HH3 and HH5 (16.74%), HH5 and CDH (12.06%), and HH6 and HH5 (11.83%).

### 3.2. Bulls

During the study, 691 bulls of the Holstein Friesian breed were examined. The frequencies of genetic defect carriers for each locus are given in Table 6. Overall, the highest carrier frequency was observed for HH5 (6.695%). No carriers of DUMPS, FXI, and HHM were detected.

## 4. Discussion

The presence of BLAD carriers in Holstein Friesian cattle has been reported in many countries such as the USA, Germany, France, Poland, Brazil, Japan, Iran, Turkey, and India. In the 1990s, bovine leukocyte adhesion deficiency (BLAD) emerged as one of the most prevalent genetic disorders within cattle populations. The genetic material from carrier bulls was integrated into the Polish bovine gene pool, primarily through the importation of semen, as well as through the importation of heifer calves from the United States and Canada during the early 1970s [8]. In Poland, since 1999, Holstein Friesian bulls of the black-and-white and red-and-white types intended for breeding have been subjected to mandatory tests for BLAD carriage. The tests were performed at the UWM in Olsztyn. The scale of BLAD carriage in Poland in 2000 reached 3.2% among bulls approved for breeding and as much as 4.6% in the mass population sample of cows. The execution of the BLAD control initiative has resulted in a discernible reduction in the prevalence of BLAD carriers within Poland. The peak prevalence of carriers of the mutant allele was documented at the inception of this initiative, reaching a level of 7.9%. Systematic population assessments have markedly diminished the risk associated with this genetic condition. Between 2004 and 2006, the prevalence of heterozygotes declined to approximately 0.8% [27]. The strategy of systematic monitoring and eradication of BLAD carriers among all young bulls purchased at the insemination station resulted in no new bull BLAD carriers being recorded in 2009 [26]. In our study, the frequency of BLAD carriers was calculated to be at the level of 0.16% for cows and 0.29% for bulls, which suggests the need for the constant monitoring of the population. BLAD has been reported to have a frequency of 0.48% in China [28]; 1.31% in Turkey [29]; and 4.0% in India [30]. On the other hand, no incidence of BLAD carriers was reported in Mexico [31,32], the Czech Republic [33], Russia [34], and India [35].

In our study, four cows were found to be carriers of DUMPS; no carriers of this defect were found among bulls. No cows were found to be carriers of DUMPS in a study carried out by Gozdek et al. [36]. Our results support the conclusions drawn by Kamiński et al. [25], who conducted an investigation involving a population of 2209 bulls and identified the absence of carriers for this mutation. The outcomes of our investigation align with the findings of other scholars who have documented the nonexistence of carriers within Holstein Friesian cattle populations across various countries, including Turkey [37,38], Russia [34], India [35], and the Czech Republic [33]. In the past, allele frequencies of genetic disorders such as DUMPS were drastically decreased by excluding carrier bulls from artificial insemination. Superior bulls were excluded from mating irrespective of their genetic merit and the frequency of genetic disorders in the population. These results may suggest that years of such breeding programs eliminated the mutant alleles from the cattle population in many countries. It would seem that the population of dairy cattle bred in Poland is free from DUMPS; however, the detection of a few DUMPS carriers shows that the population should be constantly monitored in terms of the carriage of this genetic defect.

In the present analysis, the frequency of HH1 carriers was calculated to be at the level of 2.55% for cows. Similar results were obtained by Cole et al. In 2016, they reported an HH1 frequency of 1.92% for cows [2], but in the report of 2018, this rate was 1.28% [39]. Studies performed in Russia indicate a carrier frequency of 1.83% in Holstein cows [40]. In Japanese Holstein cows, Ghanem et al. [41] found 7 carriers out of 240 Holstein cows, making the frequency of the APAF1 mutant allele 2.9%. In Chinese Holstein Friesian cows, Khan et al. [7] showed that 6.92% of cows were HH1 carriers. In our study, the frequency of HH1 carriers was calculated to be at the level of 3.18% for bulls. Kamiński [42] found 85 carriers out of 178 Polish Holstein Friesian bulls, giving a frequency of carriers of HH1 of 47.75%. This high percentage of carriers is most probably due to the small pool of animals tested. Studies performed in Russia indicate a carrier frequency of 2.04% in bulls [34]; in the Italian Holstein Friesian population, the carrier frequency in bulls is 3.42% [43]; and in Uruguay, the carrier frequency in bulls is 4.44% [44]. No carriers of HH1 were detected in Brazil [45].

Among 691 bulls, 12 HH3 carriers were found (1.74%), and among 78,870 cows, 2530 HH3 carriers were found (3.21%). Cole et al. [2] documented the frequency of HH3 at 2.95%; however, in the subsequent report of 2018, this frequency experienced a decline to 2.64% [39]. Fritz et al. [5] indicated that the frequency of HH3 exceeded 2.5% within the population of French Holstein cattle. The prevalence of lethal haplotypes was found to be 5.1% in German Holstein cattle [13], 3.13% in Uruguay’s Holstein cattle [44], and 3.0% in Kazakhstan’s Holstein cattle [46], while it stood at 5.27% in Italian Holstein cattle [43]. Zhang et al. [47] showed an HH3 carrier frequency of 2.6% among 390 cows of the Chinese Holstein variety. Similarly, Khan et al. [7] reported an HH3 frequency of 5.76% among the population of Chinese Holstein cattle.

In our study, the frequency of HH4 carriers was calculated to be 0.91% for cows and 0.87% for bulls. The HH4 haplotype carriers of cows occurred with a frequency of 3.6% in French Holstein cattle [5], 1.26% in German Holstein cattle [3], and 0.23% in American Holstein cattle [39]. Briano-Rodriguez et al. [44] reported an HH4 frequency of 1.04% in Uruguay’s Holstein bulls and 3.34% in Indian Holstein Friesian bulls [48]. Zhang et al. [47] found no carriers among 390 Chinese Holstein cows. Similarly, no cows were found to be carriers of HH4 in Chinese Holstein cattle, as reported by Khan et al. [7].

Among the cows and bulls genotyped in the present study, the highest percentage of carriers was recorded for HH5 (5.40% for cows and 6.70% for bulls). Zhang et al. [47] showed an HH5 carrier frequency of 0.8% among 390 cows of the Chinese Holstein variety. Khan et al. [7] indicated an increase in the frequency of HH5 carriers to 4.30% in the Chinese Holstein cows. Schütz et al. [13] assessed the prevalence of carriers within the examined German Holstein population to be 5.5%, while in the United States, this figure was determined to be 2.39% [39]. In the Russian context, 1202 sires and 708 Holstein cows were subjected to screening procedures. The identification of 39 bulls and 10 cows as carriers of HH5 was documented, corresponding to carrier frequencies of 3.24% and 1.41%, respectively [40]. The carrier frequency for HH5 was lower than 2% among the French Holstein cattle population, as reported in 2020 [5], and 6.88% in the Italian population [43]. The carrier percentage was 0.26% for HH5 in the Uruguay Holstein cattle population [44].

In our study, the frequency of HH6 carriers was calculated to be 2.70% for cows and 2.32% for bulls. Fritz et al. [14] conducted the comprehensive screening of 29,000 animals and identified a prevalence of 1.3% of HH6 haplotype carriers within the French Holstein population. Kamiński [49] documented the presence of 50 HH6 carriers among 87 Holstein bulls, resulting in a prevalence rate of 57.47%. This high result is most likely the result of the small pool of animals tested. In a study conducted in Russia, it was observed that the frequency of HH6 haplotype carriers within the genotyped cohort, comprising 60 cows and 63 bulls, was less than 1% [50]. Other studies of this population conducted by Zubareva et al. [51] showed a 0.95% frequency of HH6 carriers. For HH6, the carrier percentages indicated prevalences of 1.86% in cows in the Chinese Holstein Friesian cattle population [7] and 0.72% in bulls in the Italian Holstein Friesian population [43].

Among the Holstein haplotypes, our study showed the lowest percentage of carriers for HH7 (0.43%). Since HH7 is one of the newest genetic defects, detected 4 years ago, only a few reports on the frequency of carriers in Holstein Friesian cattle are available. This haplotype has a carrier frequency of 1.1% in the French Holstein cattle population [15] and 1.92% in the Russian population [51]. To our knowledge, no other reports have been published on the frequency of the HH7 haplotype.

Our study has shown the presence of significant percentage of CDH carriers in cows (2.27%). Kipp et al. [52] approximated that annually, roughly 3400 Holstein calves that are homozygous for this haplotype are born in Germany, resulting in a carrier frequency of approximately 8.7%. Schütz et al. [13] determined that the carrier frequency in Holstein cattle born in Germany from 2012 to 2015 was about 12.5%. Wang et al. [53] detected a carrier frequency of approximately 14.6% in Canadian Holstein, Ussenbekov et al. [46] reported a carrier rate of 11.0% for CDH in Kazakhstan, and Gürses and Dere [54] indicated a 4.67% rate of carriers in Turkey. Of the 1633 cows, the carrier frequency of cholesterol deficiency was 3.62% in the Chinese Holstein cattle population [7].

We identified the lowest carrier rates for FXI, mule foot, and citrullinemia. Our findings were similar to those of Khade et al. [24] and Virgen-Méndez [32], who reported that none of the animals were carriers for FXI deficiency. However, various investigators worldwide have reported mutations for FXI deficiency. Meydan et al. [55] screened 225 Holstein Friesian cows in Turkey and observed the prevalence of heterozygous cows as 8.0%. Marron et al. [18] observed the frequency of the mutated allele as 1.2% in a contemporary population of the USA Holstein sires. From India, Patel et al. [56] reported two FXI carrier Holstein Friesian bulls (frequency 0.6%). As the cited studies show, regular population monitoring led to the elimination of the FXI deficiency.

Briano-Rodriguez et al. [44] observed a frequency of mule foot of 4.18% in a contemporary population of Holstein Friesian cows in Uruguay. Chao et al. [57] found that no carriers of HHM were detected in reported in Taiwan.

Citrullinemia was one of the earliest known genetic defects. Numerous attempts have been made to eliminate carriers of this genetic disorder. Currently, the frequency of the mutant allele is either very low or there are no carriers of this trait in Holstein Friesian populations [58].

There are many methods to combat the spread of these genetic defects. In the past, all bulls carrying a given genetic disease were excluded from mating, irrespective of their superior genetic merit and the frequency of the genetic disorder in the population. Such a strategy leads to lower genetic gain for production and functional traits [59]. Unfortunately, cows were not frequently tested in the past.

For each genetic defect, comprehensive investigation, including an assessment of allele frequencies, inheritance mode, economic value, and causal mutation, is needed to find the best way of handling the genetic characteristics in breeding programs. In most cases, the carrier frequency can be managed by finding the appropriate mating partner (e.g., mating a carrier sire only with noncarrier dams). An example by VanRaden et al. [60] showed that the famous key ancestor Pawnee Farm Arlinda Chief carried the HH1 haplotype. This sire contributed 14% of his gene variants to the current Holstein population, which increased the milk yield by $25 billion. In contrast, the costs for mid-term abortions due to HH1 were only $0.4 billion [3].

For mating decisions, it is necessary to take into account not only the carrier state of genetic defects but also economically beneficial genetic characteristics such as polledness and milk proteins. This approach will reduce the number of genetic disorders, and subsequently may have implications for genetic gain in terms of fertility traits, animal welfare, and the overall image of the breed. In addition, economically advantageous traits such as polledness should be expanded in dairy populations to avoid the dehorning of young calves.

The expenditure associated with a particular defect depends on the carrier frequency, the time of the abortion, or the time of death. The financial loss attributable to recessive haplotypes was estimated at 7.5 million US dollars in the American dairy industry in 2016 [2]. It has been estimated that the economic impact of spontaneous abortion attributed to HH1 is approximately 140,000 USD in the United States and around 525,000 USD on a global scale, cumulatively resulting in a financial loss of $420 million [61].

The management of the genetic disorders with lower frequencies, like HH6, HH7, or DUMPS, is relatively easy and involves avoiding carrier mating and gradually reducing the number of carriers among breeding bulls [15]. Although avoiding carrier-to-carrier mating is a simple way to eliminate carriers from the population, an increasing number of identified defects make this difficult [62].

Cole et al. [39] proposed a different approach in which individuals’ estimated genetic merit is adjusted for economic losses due to fatal losses. This approach effectively reduces the prevalence of recessive lethal alleles while concurrently preserving existing rates of genetic improvement. A heterozygous carrier animal may be utilized for breeding purposes if its advantageous alleles can compensate for the losses incurred due to lethal alleles.

## 5. Conclusions

Our study represents an important attempt to extensively screen the occurrence of selected genetic defects among Holstein Friesian cattle in Poland. The occurrence of these defects among Polish dairy cattle has never been extensively screened before. Our findings reveal a fairly high frequency of carriers of some genetic defects in the Polish Holstein Friesian population (HH5, HH3, HH1), and their frequency may increase if no action is undertaken. The high carrier frequency of these genetic defects indicates an urgent need for routine molecular testing to eliminate the deleterious alleles. The population of Holstein Friesian cows in Poland is approximately 2.4 million. Therefore, the strategy of limiting the number of carriers is desirable to reduce losses in the fertility and profitability of dairy cattle production in the future. Knowledge about the occurrence of genetic defects in the herd (both cows and bulls) is of significant importance in breeding programs. It helps breeders to make appropriate mating decisions. In Poland, PFCB&DF introduced a tool in the DoKo software version 4.5F (2024.2) API (animal mating algorithm) in May 2022, intended to reduce the mating of animals that are carriers of the same genetic defect. Thanks to this solution, it is possible to decrease the number of carriers of genetic defects and, thus, economic losses for breeders. However, the complete culling of carriers of lethal recessive alleles is not practical due to their high overall prevalence and the increasing number of identified mutations. An appropriate strategy for herd management would lead to avoiding economic losses and a decreased frequency of mutant alleles in the cattle population.

## Figures and Tables

**Table 1:** Basic characteristics of selected genetic disorders.

Defect	Name	Gene	OMIA	Year Published
HH1	Holstein haplotype 1	*APAF1*	000001-9913	2016
HH3	Holstein haplotype 3	*SMC2*	001824-9913	2014
HH4	Holstein haplotype 4	*GART*	001826–9913	2013
HH5	Holstein haplotype 5	*TFB1M*	001941-9913	2016
HH6	Holstein haplotype 6	*SDE2*	002149-9913	2018
HH7	Holstein haplotype 7	*CENPU*	001830-9913	2020
BLAD	Bovine leukocyte adhesion deficiency	*ITGB2*	000595-9913	1992
DUMPS	Deficiency of uridine monophosphate synthase	*UMPS*	000262-9913	1993
CDH	Holstein cholesterol deficiency	*APOB*	001965-9913	2016
FXI	Factor XI deficiency	*F11*	000363-9913	2004
HHM	Syndactyly (mule foot)	*LRP4*	000963-9913	2006
BC	Citrullinaemia	*ASS1*	000194-9913	1989

**Table 2:** The number of animals genotyped on individual microarrays.

Microarrays	Number of Cows	Number of Bulls
EuroGenomics_MD_v4-1_POL	42,263	321
EuroGenomics_MD_v4_POL	15,766	253
EuroGenomics_MD_v3_POL	13,195	117
EuroGenomics_MD_v2_POL	7660	0

**Table 3:** Single nucleotide polymorphism (SNP) information.

Defect	SNP Name	Variant Description		
		Chr	Description	Coding DNA Change
HH1	EuroG10K_HH1	5	nonsense	c.1741C>T
HH3	EuroG10K_HH3_SMC2	8	missense	c.3404T>C
HH4	EuroG10K_HH4_GART	1	missense	c.869A>C
HH5	EuroG10K_TFB1M_DEL_HH5_R_B	9	gross deletion	138 kb deletion
HH6	EGX_HH6_SDE2	16	start-lost	c.2T>C
HH7	EGX_HH7_CENPU	27	small deletion	c.15123637_15123640delTTACT
BLAD	ITGB2	1	missense	c.383A>G
DUMPS	EuroG10K_SNP_AF368419_1247	1	nonsense	c.1213C>T
CDH	EuroG10K_ERV_11_77958995_77959000_R	11	gross insertion	1.3 kb insertion
FXI	EuroG10K_FXI_WA	27	gross insertion	76 bp insertion
HHM	LRP4_5	15	small delins	c.4863_4864delinsAT
Citrullinaemia	ASS1	11	nonsense	c.256C>T

**Table 4:** Frequencies of genetic defect carrier cows.

Haplotype	Number of Genotyped Cows ^1^	Number of Carriers	Percentage of Carriers (%)
HH5	78,621	4249	5.40
HH3	78,870	2530	3.21
HH6	78,873	2132	2.70
HH1	78,866	2014	2.55
CDH	78,883	1792	2.27
HH4	78,884	721	0.91
HH7	78,884	205	0.26
BLAD	78,876	128	0.16
HHM	78,883	15	<0.01
DUMPS	78,882	4	<0.01
BC	78,883	2	<0.01
FXI	78,882	1	<0.01

^1^ in some defects, the probe was not read and therefore the number of animals was lower than 78,884.

**Table 5:** Number of cows carrying single or multiple genetic defects.

	Number of Cows	Percentage
Single defect	12,927	16.39%
Double defects	854	1.08%
Triple defects	11	0.01%
Quadruple defects	1	<0.01%
Overall	13,793	17.48%

**Table 6:** Frequencies of genetic defect carrier bulls.

Haplotype	Number of Genotyped Bulls	Number of Carriers	Percentage of Carriers (%)
HH5	687	46	6.70
HH1	691	22	3.18
HH6	691	16	2.32
HH3	691	12	1.74
HH4	691	6	0.87
CDH	691	6	0.87
HH7	691	3	0.43
BLAD	691	2	0.29
BC	691	1	0.15
DUMPS	691	0	0.00
FXI	691	0	0.00
HHM	691	0	0.00

## Data Availability

Data are contained within the article.
